# COVID-19 Pneumonia in Children: From Etiology to Management

**DOI:** 10.3389/fped.2020.616622

**Published:** 2020-12-14

**Authors:** Giuseppe Fabio Parisi, Cristiana Indolfi, Fabio Decimo, Salvatore Leonardi, Michele Miraglia del Giudice

**Affiliations:** ^1^Department of Clinical and Experimental Medicine, University of Catania, Catania, Italy; ^2^Department of Woman, Child and Specialized Surgery, University of Campania “Luigi Vanvitelli”, Naples, Italy

**Keywords:** COVID-19, children, pneumonia, SARS-CoV2, treatment

## Abstract

COVID-19 is less serious in children than in adults. However, respiratory management dominates the clinical picture of hospitalized COVID-19 even in children. In some case series, deterioration of the clinical picture wherein dyspnea, cyanosis, and the onset of acute respiratory distress syndrome (ARDS) emerged ~8–10 days after the onset of SARS-CoV-2 infection, which could rapidly progress to multiple organ failure and death. This review aimed to evaluate the characteristics of COVID-19 pneumonia in pediatric populations, beginning from its etiology and pathological mechanisms and closing with its clinical management.

## Introduction

At the close of December 2019, a new coronavirus originating from the Chinese city of Wuhan began to spread rapidly throughout the world (1). At the beginning of 2020, the International Committee on Taxonomy of Viruses denominated this new virus Severe Acute Respiratory Syndrome Coronavirus 2 (SARS-CoV-2) (2). SARS-CoV-2 is the causative agent of the disease COVID-19, an abbreviation decided by the World Health Organization (WHO). In other words, SARS-CoV-2 is the etiologic agent, while COVID-19 is the disease (3).

The clinical spectrum of COVID-19 is wide, varying from completely asymptomatic forms to those characterized by severe respiratory distress requiring intensive care. SARS-CoV-2 causes acute viral infection of both the upper and lower respiratory tract, with an incubation period varying from 1 to 15 days (average: 3–7 days). The most common symptoms of COVID-19 include fever, cough, sore throat, headache, asthenia, diarrhea, and vomiting (4).

There is ample evidence in the literature that COVID-19 is less serious in children than in adults (5–10). Lu et al. found the main symptoms in 171 children with COVID-19 to be cough (48.5%), pharyngitis (46.2%), fever (41.5%), diarrhea (8.8%), and vomiting (6.4%); only 2.3% of cases experienced desaturation upon hospitalization, while 15.8% of cases were asymptomatic (9). Olfactory and gustatory anomalies characteristic of adult COVID-19 cases are rare in pediatric populations (11, 12).

The underlying cause of the lower incidence and pathogenicity of SARS-CoV-2 infection in children remains unclear at present. Although this lower incidence and morbidity was attributed to a reduced exposure and the presence of risk factors during the initial phase of the pandemic, it is now clear that biological factors that intervene in the pathogenesis of the infection and in the immune response may play a protective role in children against the more aggressive clinical manifestations seen in adults (13).

Respiratory management dominates the clinical picture of hospitalized COVID-19 patients. In some case series, deterioration of the clinical picture wherein dyspnea, cyanosis, and the onset of acute respiratory distress syndrome (ARDS) emerged approximately 8–10 days after the onset of SARS-CoV-2 infection, which could rapidly progress to multiple organ failure and death (14). In a pediatric series of children with COVID-19, 30.8% presented shortness of breath that required oxygen supplementation and 23.1% were transferred to intensive care unit (ICU) for organ dysfunction (15). In another case series of 41 children hospitalized for COVID-19, 11 of these presented lung lesions compatible with a picture of interstitial pneumonia (16). Furthermore, in one of the largest published pediatric series that studied 585 children with SARS-CoV2 infection, 8% required ICU admission and 4% needed mechanical ventilation (17).

Although the clinical picture in pediatric populations is more complex, the severity of infection can be clinically classified as follows: asymptomatic, mild, moderate, severe, or critical (18, 19) (Table 1). This classification makes the idea that even pediatric patients can experience severe manifestations of the pathology, which must be addressed as early as possible to limit disease progression.

**Table 1:** Classification of COVID-19 in children.

**Classification**	**Clinical features**
Asymptomatic	Positivity of the RT-PCR buffer to SARS-CoV-2 or positive serology in the absence of any symptoms of illness.
Mild	Symptoms are mild and mainly affect the upper airways (nasal obstruction, sneezing) sometimes associated with fever, cough, and gastrointestinal symptoms.
Moderate	Symptoms are more critical fever and cough (mainly dry) are almost always present and are associated with breathing difficulties. It is characterized radiologically by lung anomalies compatible with interstitial pneumonia.
Severe	It is characterized by the presence of hypoxemia (SpO_2_ <92%) with signs of respiratory distress (tachypnea, groaning, wing flaps, sags), cyanosis, neurological signs and symptoms, refusal to eat, and signs of dehydration.
Critical	Disease progression with onset of respiratory failure requiring mechanical ventilation, signs of shock or multi-organ failure.

This review aimed to evaluate the characteristics of COVID-19 pneumonia in pediatric populations, beginning from its etiology and pathological mechanisms and closing with its clinical management.

## Methods

This review used PubMed and Science Direct to locate articles with at least an English abstract using the following keywords: (1) COVID-19 in children; (2) coronavirus in children; (3) COVID-19 pneumonia; (4) SARS-CoV-2 in children; (5) SARS-CoV-2 pneumonia; and (6) COVID-19 imaging. The abstracts of articles were reviewed to determine whether the article was appropriate for the topic. We also reviewed the references contained within the selected articles, and read the full articles that were deemed relevant.

## Epidemiology

The SARS-CoV-2 virus is transmitted via droplets and through direct or indirect contact with infected objects (1). The time during which the virus remains active on surfaces remains unclear, but was found to be ~48–72 h on plastic and steel, and ~4–8 h on copper and cardboard (20). Cohabitation with symptomatic or asymptomatic patients is the main source of contagion for pediatric populations (21), but given the frequency of paucisymptomatic forms in pediatric populations, children are likely to be a frequent vector of infection for adults and the elderly. The positivity in reverse transcription polymerase chain reaction (RT-PCR) for SARS-CoV-2 in the stools of infants and children for several weeks, even after a negative nasopharyngeal swab (22), may indicate the stools could represent an additional means of transmission of the virus.

However, since growth of the virus in fecal culture—and therefore its viability on feces—has not been demonstrated, further research is needed to define a possible fecal–oral route of transmissibility of the virus. Similarly, maternal–fetal transmissibility of the virus has been explored since the beginning of the epidemic. A first report on nine women with COVID-19 in their third trimester of pregnancy confirmed the absence of SARS-CoV-2 in amniotic fluid, cord blood, and breast milk (23). More recently, the maternal–fetal transmission has been confirmed in three infants (transmission rate: 9%) born to a positive mother; one of these infants had onset of respiratory symptoms within 48 h of life (24). However, a larger retrospective cohort analysis involving 101 infants born to 100 SARS-CoV2 positive mothers did not show vertical transmission in any of these (25).

## Pathogenesis of Lung Damage

**Figure 1:**
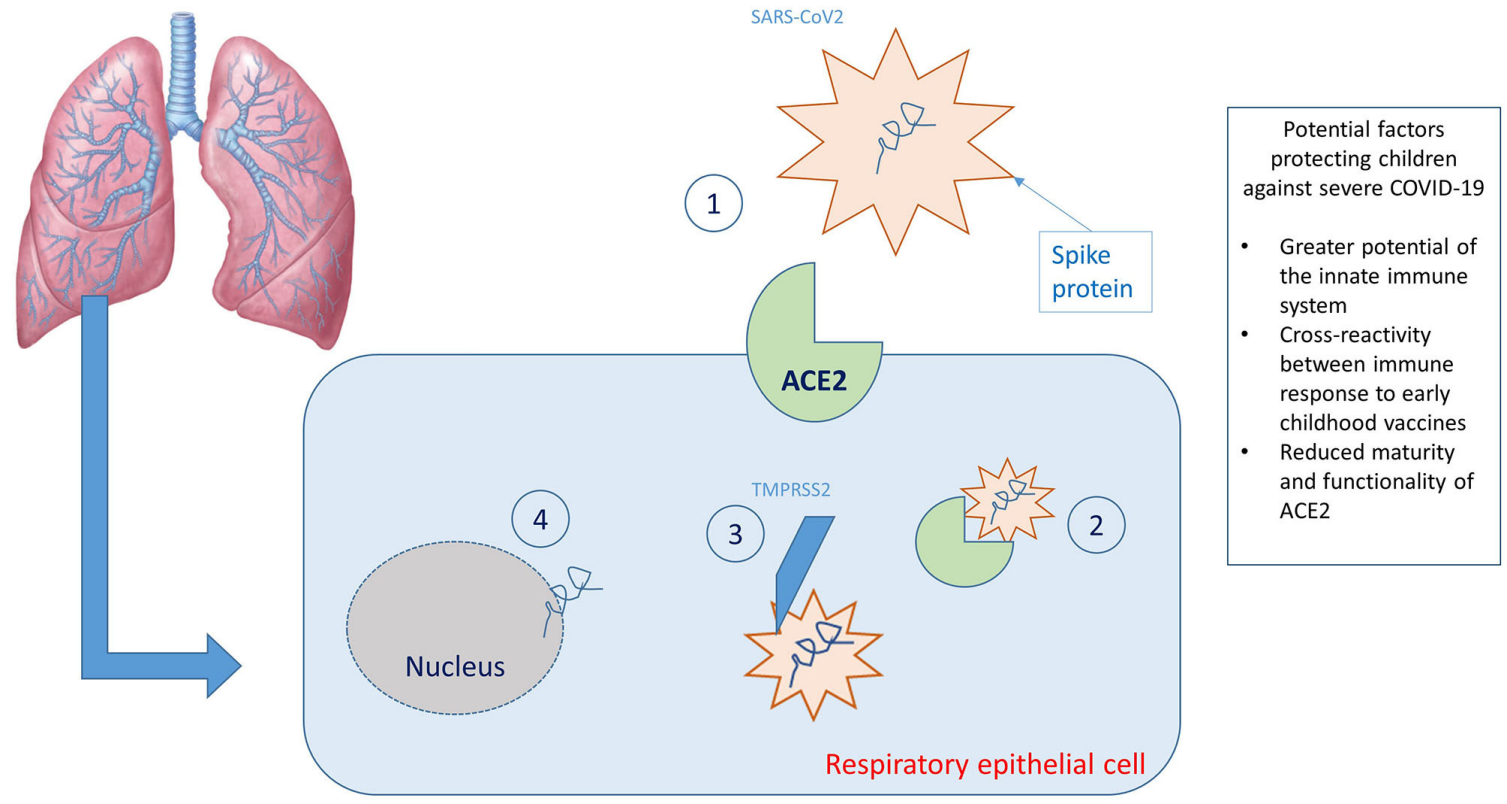
Graphic representation of the virus-host interaction and reasons why children are less affected. (1) Binding between SARS-CoV2 spike protein and ACE2 receptors; (2) transport of the ACE2 receptor/SARS-CoV 2 complex within the cell; (3) cleavage of the spike protein by the protease TMPRSS2; (4) release of viral RNA within the cell.

When SARS-CoV-2 enters the airways of a newly infected person, the viral S protein (spike protein) binds with high affinity to the angiotensin-converting enzyme 2 (ACE2) cellular transmembrane receptor found on the apical membranes of respiratory epithelial cells, mainly type II pneumocytes. Subsequently, the ACE2 receptor and SARS-CoV-2 are transported inside the cell and the S protein is cleaved by the protease TMPRSS2, inducing the release of the viral RNA within the cell and thereby allowing its replication (Figure 1). The ACE2 receptor is subsequently cleaved by a tumor necrosis factor alpha converting enzyme (TACE or ADAM17), a metalloprotease that allows the release of the ACE2 ectodomain (defined as soluble ACE2) into the extracellular space. Soluble ACE2 is enzymatically active and appears to be capable of binding with SARS-CoV-2. This led to speculation that administration of recombinant human ACE2 may reduce inflammation secondary to the action of SARS-CoV-2 (26).

The immune response induced by SARS-CoV-2 infection is characterized by two phases: an initial immunoprotective phase and an activation phase of the cytokine storm, which yields a more severe clinical manifestation. In the first phase, a robust adaptive response can control the virus and block inflammatory progression. If the immune system fails to control this phase, cell damage in organs with high concentrations of ACE2, especially pneumocytes, progresses by the release of cytokines and chemokines (IL-6, IL-10, and interferon) and the recruitment of inflammatory cells, which mediate lung damage and progression toward ARDS (27). Xu et al. found evidence of diffuse alveolar damage with desquamation of pneumocytes, hyaline membrane formation, and the presence of fibromyxoid cells with interstitial lymphocyte infiltration during histopathological examination of a patient who died of COVID-19 (28). In fact, clinically speaking, SARS-CoV-2 causes interstitial pneumonia.

One of the possible complications of this “exaggerated” inflammation is Pediatric Inflammatory Multisystem Syndrome (PIMS) or Multisystem Inflammatory Syndrome of Children (MIS-C) which occurs when inflammation becomes generalized. This would appear to be a post-immunological reaction caused by non-neutralizing IgG antibody and worsened by a cytokine storm that causes generalized inflammation that resembles an atypical Kawasaki's disease or a toxic shock syndrome (29).

### Why Are Children Less Affected?

Many explanations have been proposed for the fact that children appear to be less frequently affected and have milder manifestations of COVID-19; however, they remain assumptions given the lack of scientific evidence on the subject.

The immune response of children differs from that of adults, which progressively deteriorates with age such that preschoolers have a repertoire of immune cells 5–10 times larger than that of a 50-year-old, and 20 times larger than that of an 80-year-old. It remains to be seen to what degree this may play a role in mitigating the spread of the virus and in the cytokine signaling cascade triggered by SARS-CoV-2 as they relate to severe outcomes in adulthood (30).

A cross-reactivity between immune response to early childhood vaccines—especially MMR—and response to SARS-CoV-2 has also been proposed. However, no clear evidence has emerged to date to support this proposal, and paucisymptomatic cases are reported even in unvaccinated children. A large pediatric clinical series on 2,143 children reported a 5.9% rate of serious and critical cases—and only one death (31). Shekerdemian et al. reported a mortality rate of 4.2% in a cohort of 48 COVID-19-positive children admitted to the ICU, most of whom had previous comorbidities (disabling genetic diseases in 40% of cases) (32).

A second explanation for the tendency of children be less affected by SARS-CoV-2 relates to the ACE2 receptor which, as previously mentioned, binds the SARS-CoV-2 virus. In fact, the reduced maturity and functionality of ACE2 and its lower expression in the nasal epithelium in pediatric populations relative to adults could partly explain children's reduced susceptibility to COVID-19 (33, 34). However, studies do not unilaterally support this hypothesis. In fact, some evidence suggests that ACE2 expression in children is neither up- nor downregulated (35, 36). On the other hand, another study found that ACE2 is downregulated once the virus penetrates the cell and replicates, resulting in fewer receptors upon which the virus can act (37). In light of these data, the role of the expression levels of ACE2 in the pathogenesis of COVID-19-induced lung damage remains to be fully elucidated.

## Chest Imaging

According to the American College of Radiology, pediatric radiologic imaging is recommended for patients with a confirmed diagnosis of COVID-19 with mild symptoms and pre-existing comorbidities, and for children with moderate to severe symptoms. Chest X-ray is the first choice exam; however, its lower sensitivity and specificity mean that pulmonary involvement cannot be excluded in patients with a laboratory-confirmed diagnosis of COVID-19. Unlike in adults, computed tomography (CT), is indicated in children in cases of suspicion of pulmonary embolism and clinical worsening (38).

### Chest X-Ray

Pulmonary abnormalities on chest X-ray were found in 46–90% of cases (39, 40). The most common radiologic trends were peribronchial thickening, ground-glass opacities, consolidation, and pleural effusion (39). Lung findings were unilateral in 55% and bilateral in 45% of affected children, without any significant difference between the left and right lung, but with greater involvement of the lower lobes (40). Although these radiological abnormalities typically resolve during recovery from the disease, they have been reported to persist in ~16% of cases (39).

### Chest Computed Tomography (CT)

The radiological anomalies evident on CT are certainly much more characterizing the disease, although these anomalies are less frequent and less specific than those described in adults (41). The most common findings are multifocal and peripherally located ground-glass appearance starting from the lower lobes, accompanied by thickening in the interlobular septa, prominent vascular structures, halo signs, and inverted halo signs. In severe cases, a striking paving appearance and fragmented consolidation are observed (40, 41).

Liu et al. described the radiological characteristics examined by high-resolution (HR)-CT of five children with a confirmed diagnosis of COVID-19, three of whom were asymptomatic. These three patients had unilateral ground glass opacities, whereas only one child had bilaterally distributed opacity, and another had a negative report (42). Some radiological differences between children and adults were highlighted (43). Although the ground glass finding is characteristic and common in both adulthood and childhood, 44% of adult patients also had thickening of the interlobular septum, bronchial texture, a striking paving pattern, and—less frequently—halo signs, pleural or pericardial effusion, and lymphadenopathy (44).

In a group of 98 patients of varying ages (4–88 years) with COVID-19, the majority of lung lesions upon HR-CT were located in the right lower lobe of the lung, possibly due to the thinner and shorter structure of the lower lobe bronchus, especially in the peripheral area of the lung. However, children and adolescents had fewer lung lesions, predominantly unilateral involvement, and smaller clusters than adults, with no signs of air bronchogram (45). Zheng et al. also reported a higher incidence of respiratory impairment in children <3 years of age, with bilateral lung involvement in >70% of children in this age group (46). A report of eight patients aged <15 years admitted to intensive care found abnormalities on CT scans in all cases (six children with bilateral involvement, two with unilateral involvement). Moreover, two of the eight patients that originally had a worse prognosis also had higher expression of IL-6 and IL-10, further corroborating a relationship between the severity of the pulmonary picture and activation of the cytokine cascade (47).

### Lung Ultrasound

Several studies report the usefulness of pulmonary ultrasound for the diagnosis and follow-up of COVID-19 pneumonia, given that it is a simple and repeatable investigation that does not expose the child to radiation or sedation. Musolino et al. reported the main ultrasound findings of 10 children with COVID-19 as follows: B lines (70%), pleural irregularities (60%), white lung (10%), and subpleural thickening (10%) (48, 49). According to Allinovi et al., lung ultrasound may support diagnosis and monitoring of COVID-19 pneumonia, as it reveals a typical pattern of diffuse interstitial lung syndrome and correlates with chest CT findings (50).

## Management and Treatment of COVID-19 Pneumonia

Given the paucisymptomatic course characteristic of children diagnosed with COVID-19, the majority of cases only require supportive home therapy. Evidently, cases must be isolated, and they require an adequate intake of fluids and calories (51, 52). For the management of fever, paracetamol is recommended. Some authors have proposed a correlation between the use of ibuprofen and a more aggressive course of SARS-CoV-2 infection (53); however, these data were not confirmed. For patients already being treated with topical steroids (e.g., for allergic rhinitis or bronchial asthma), continuation of basic therapy is indicated. In case of need for inhalation treatment with steroids and bronchodilators, the use of pressurized metered-dose inhalers with spacer is recommended over nebulizers, which could increase infectivity due to their aerosolization of particles (54).

Hospitalization is indicated when there is a need to ensure supportive therapy (e.g., pharmacological or respiratory support) or in severe forms of pathology (13, 19, 55). Upon entering the ward, performing laboratory blood testing may prove useful, even though it is often non-specific. In most children it is possible to find: (i) a normal or reduced number of white blood cells, accompanied by lymphocytopenia; (ii) normal or slightly increased C-reactive protein and procalcitonin values (in case of excessive upregulation, a bacterial superinfection should be considered); (iii) slightly increased transaminases and lactic dehydrogenases (13, 19, 55).

Patients with chronic diseases should be subjected to greater attention because the presence of comorbidities seems to be associated with a greater risk of fatal evolution (56). In this sense, these patients should be monitored more frequently and subjected to earlier treatments.

### General Support

Hospitalized children must have their vitals monitored and have adequate intake of fluids and calories aimed at maintaining a hydro-electrolytic homeostasis. Additionally, bed rest and maintenance of cleared upper airways are recommended (13, 19, 55).

### Oxygen Therapy

In case of hypoxia (SpO_2_ <95%) without signs of respiratory distress, the administration of oxygen via nasal cannulae or mask is sufficient, while constant monitoring of vital parameters and attending to changes in the acid-base balance may be indicative of clinical worsening (13, 19, 55).

### Ventilatory Support

In case of respiratory distress associated with hypoxemia, simple oxygen administration is insufficient. In these cases, high-flow nasal oxygen (HFNO) or non-invasive ventilation, such as continuous positive airway pressure (CPAP), should be used (13, 19, 55). The utility of HFNO for COVID-19 treatment is the subject of debate given that the incontrovertible benefits afforded by this treatment are countered by the risk of viral particle aerosolisation within the patient's environment, thereby placing the safety of healthcare workers at risk (57). The World Health Organization (WHO) recommends that HFNO be used in single or negative pressure rooms “whenever possible.” This means that negative pressure room, while advantageous, are not essential (58). What is certainly essential, however, is the use of personal protective equipment (PPE) when entering patients' rooms (57).

A valid alternative to HFNO is CPAP—preferably helmet CPAP—with positive end-expiration pressure (PEEP) ranging from 5 to 10 cmH_2_0 (59). In any case, the critically ill child should be transferred to a pediatric intensive care unit and, in the event of non-response to non-invasive ventilation or of onset of pediatric acute respiratory distress syndrome (PARDS), initiation of invasive mechanical ventilation should be considered and, ultimately, extracorporeal membrane oxygenation (ECMO) (19).

### Pharmacological Treatment

There is little reliable evidence for the utility of drugs in treating COVID-19 pneumonia in pediatric populations, and any available data to date are based on observations in adult populations. For this reason, pharmacological therapy discouraged in milder COVID-19 forms, while recommended for more severe forms; such decisions should invariably be made on a case-by-case basis (13, 19, 51, 55, 60).

No specific anti-SARS-CoV-2 drug has yet been proven effective. Antiviral drug therapy seems to be effective if initiated before clinical deterioration. The drug most commonly used is interferon-alpha by nebulization, as it has shown effectiveness at reducing viral replication with consequent improvement of symptoms and reduction of the duration of the disease (21). Other possible pharmacological interventions include:

- Lopinavir/Ritonavir: A drug used in the treatment of HIV which appears to be effective in reducing viral replication as long as it is administered in the very early stages of the disease. Common side-effects include diarrhea and nausea, and it is contraindicated in cases of hepatic impairment (61).
- Ribavirin: A drug used in combination with interferon-alpha or Lopnavir/Ritonavir. Hemolytic anemia is a possible side-effect (19, 62).
- Remdesevir: A new-generation antiviral that has a potent antireplicative action against SARS-CoV-2 (63, 64).
- Hydroxychloroquine: A drug that, despite the initial enthusiasm surrounding its use for treatment of COVID-19, has not shown real efficacy according to the most recent scientific evidence (65). Table 2 summarizes the main antivirals, their formulations, and their respective dosages in pediatric patients.

**Table 2:** Summary of most common antivirals for COVID-19 in children.

**Antiviral**	**Route of administration**	**Pediatric dose**	**Duration of treatment**
Interferon-α^*^	Inhalation	200,000–400,000 IU/kg in 2 mL of sterile water, twice daily	5–7 days
Lopinavir/Ritonavir	Oral	12 mg/3 mg/kg if weight 7–15 kg, 10 mg/2.5 mg/kg if weight 15–40 kg, 400 mg/100 mg (adult dose) if weight > 40 twice daily	1–2 weeks
Ribavirin	Intravenous	10 mg/kg/dose, 2 or 3 times daily	Max 5 days
Remdesevir	Intravenous	5 mg/kg loading dose, then 2.5 mg/kg once daily	10 days
Hydroxychloroquine sulfate	Intravenous	3–5 mg/kg/day (max dose 400 mg), twice daily	5 days

* *Most commonly used*.

Other drugs worth mentioning include:

- Antibiotics: Their use is discouraged unless there are signs of bacterial co-infection. The usefulness of macrolides, especially azithromycin, for their anti-inflammatory properties is also questionable (19, 52, 60, 66).
- Corticosteroids: Their routine use is discouraged; however, they should be considered in cases of PARDS, secondary haemophagocytic lymphoistiocytosis, septic shock, or concomitant asthma. In these cases, the administration of methylpredisolone at a dose of 1–2 mg/kg/day for a maximum of 4–5 days is recommended (19, 52, 60).
- Gamma globulins: Their effectiveness is not clear. They can be attempted in particularly severe forms of COVID-19 and in those with symptoms similar to Kawasaki disease at the dose of 2 g/kg/day for one day, 1 g/kg/day for two days or 400 mg/kg/day for five days (67, 68).
- Tocilizumab: This human anti-IL-6 monoclonal antibody appeared to be effective in the treatment in adults with extensive and bilateral lung involvement (60). However, recently its effectiveness has been greatly diminished to the extent that it appears not effective for preventing intubation or death in moderately ill hospitalized patients with COVID-19 (69). For this reason, it should be used cautiously in children: 12 mg/kg in children weighing <30 kg, 8 mg/kg (max: 800 mg) in children >30 kg, to repeat once after 12 h if no improvement (60, 70).

## Conclusion

This review summarizes the characteristics of COVID-19 in pediatric populations, with a focus on pulmonary involvement. Although clinical picture of COVID-19 in children is much less severe than in adults, progression of the disease remains possible and must, therefore, be intercepted with appropriate therapy. It should also be emphasized that children, although paucisymptomatic, are important vectors of the disease.

## Author Contributions

MM developed the original idea and made the final revision. GP wrote the manuscript. CI and FD revised the manuscript and contributed to the English revision and compilation of references. SL made the final analysis and critical revision of the manuscript. All authors contributed to the article and approved the submitted version.

## Conflict of Interest

The authors declare that the research was conducted in the absence of any commercial or financial relationships that could be construed as a potential conflict of interest.

## References

[1] Zhu N Zhang D Wang W Li X Yang B Song J China novel coronavirus investigating and research team. A Novel Coronavirus from Patients with Pneumonia in China, 2019. N Engl J Med. (2020) 382:727–33. 10.1056/NEJMoa2001017 31978945 PMC7092803

[2] International Committee on Taxonomy Viruses Naming the 2019 Coronavirus (2020). Available online at: https://talk.ictvonline.org/ (accessed October 11, 2020).

[3] World Health Organization Naming the Coronavirus Disease (COVID-19) and the Virus That Causes It (2020). Available online at: https://www.who.int/emergencies/diseases/novel-coronavirus-2019/technical-guidance/naming-the-coronavirus-disease-(covid-2019)-and-the-virus-that-causes-it (accessed October 11, 2020).

[4] Guan WJ Ni ZY Hu Y Liang WH Ou CQ He JX Clinical characteristics of coronavirus disease 2019 in China. N Engl J Med. (2020) 382:1708–20. 10.1056/NEJMoa2002032 32109013 PMC7092819

[5] Ludvigsson JF. Systematic review of COVID-19 in children shows milder cases and a better prognosis than adults. Acta Paediatr. (2020) 109:1088–95. 10.1111/apa.15270 32202343 PMC7228328

[6] Zimmermann P Curtis N. Coronavirus infections in children including COVID-19: an overview of the epidemiology, clinical features, diagnosis, treatment and prevention options in children. Pediatr Infect Dis J. (2020) 39:355–68. 10.1097/INF.0000000000002660 32310621 PMC7158880

[7] Ong JS Tosoni A Kim YJ Kissoon N Murthy S. Coronavirus disease 2019 in critically ill children: a narrative review of the literature. Pediatr Crit Care Med. (2020) 21:662–6. 10.1097/PCC.0000000000002376 32265372 PMC7176259

[8] Liguoro I Pilotto C Bonanni M Ferrari ME Pusiol A Nocerino A. SARS-COV-2 infection in children and newborns: a systematic review. Eur J Pediatr. (2020) 179:1029–46. 10.1007/s00431-020-03684-7 32424745 PMC7234446

[9] Lu X Zhang L Du H Zhang J Li YY Qu J SARS-CoV-2 infection in children. New Engl J Med. (2020) 382:1663–5. 10.1056/NEJMc2005073 32187458 PMC7121177

[10] Castagnoli R Votto M Licari A Brambilla I Bruno R Perlini S. Severe acute respiratory syndrome coronavirus 2 (SARS-CoV-2) infection in children and adolescents: a systematic review. JAMA Pediatr. (2020) 174:882–9. 10.1001/jamapediatrics.2020.1467 32320004

[11] Parisi GF Brindisi G Indolfi C Diaferio L Marchese G Ghiglioni DG. Upper airway involvement in pediatric COVID-19. Pediatr Allergy Immunol. (2020) 31(Suppl 26):85–8. 10.1111/pai.13356 33236430 PMC7753446

[12] Diaferio L Parisi GF Brindisi G Indolfi C Marchese G Ghiglioni DG. Cross-sectional survey on impact of paediatric COVID-19 among Italian paediatricians: report from the SIAIP rhino-sinusitis and conjunctivitis committee. Ital J Pediatr. (2020) 46:146. 10.1186/s13052-020-00906-4 33023616 PMC7538039

[13] Zardini H Soltaninejad H Ferdosian F Hamidieh AA Memarpoor-Yazdi M. Coronavirus Disease 2019 (COVID-19) in children: prevalence, diagnosis, clinical symptoms, and treatment. Int J Gen Med. (2020) 13:477–82. 10.2147/IJGM.S262098 32848446 PMC7425102

[14] Huang C Wang Y Li X Ren L Zhao J Hu Y. Clinical features of patients infected with 2019 novel coronavirus in Wuhan, China. Lancet. (2020) 395:497–506. 10.1016/S0140-6736(20)30183-5 31986264 PMC7159299

[15] Cai X Jiang H Zhang S Xia S Du W Ma Y. Clinical manifestations and pathogen characteristics in children admitted for suspected COVID-19. Front Med. (2020) 1–10. 10.1007/s11684-020-0820-7.[Epub ahead of print]. 33106939 PMC7587538

[16] Zhang Y Xie RM He YL Xing LH Dong L Zhang JZ. Clinical and imaging features of pediatric COVID-19. Ital J Pediatr. (2020) 46:153. 10.1186/s13052-020-00917-1 33054802 PMC7556551

[17] Götzinger F Santiago-García B Noguera-Julián A Lanaspa M Lancella L Calò. COVID-19 in children and adolescents in Europe: a multinational, multicentre cohort study. Lancet Child Adolesc Health. (2020) 4:653–61. 10.1016/S2352-4642(20)30177-2 32593339 PMC7316447

[18] Chen ZM Fu JF Shu Q Chen YH Hua CZ Li FB. Diagnosis and treatment recommendations for pediatric respiratory infection caused by the 2019 novel coronavirus. World J Pediatr. (2020) 16:240–6. 10.1007/s12519-020-00345-5 32026148 PMC7091166

[19] Miao H Li H Yao Y Wu M Lu C Wang J. Update on recommendations for the diagnosis and treatment of SARS-CoV-2 infection in children. Eur J Clin Microbiol Infect Dis. (2020) 39:2211–23. 10.1007/s10096-020-03973-x 32761481 PMC7406700

[20] van Doremalen N Bushmaker T Morris DH Holbrook MG Gamble A Williamson BN. Aerosol and surface stability of SARS-CoV-2 as compared with SARS-CoV-1. N Engl J Med. (2020) 382:1564–7. 10.1056/NEJMc2004973 32182409 PMC7121658

[21] Shen K Yang Y Wang T Zhao D Jiang Y Jin R Diagnosis, treatment, and prevention of 2019 novel coronavirus infection in children: experts' consensus statement. World J Pediatr. (2020) 16:223–31. 10.1007/s12519-020-00343-7 32034659 PMC7090771

[22] Xu Y Li X Zhu B Liang H Fang C Gong Y. Characteristics of pediatric SARS-CoV-2 infection and potential evidence for persistent fecal viral shedding. Nat Med. (2020) 26:502–5. 10.1038/s41591-020-0817-4 32284613 PMC7095102

[23] Chen H Guo J Wang C Luo F Yu X Zhang W. Clinical characteristics and intrauterine vertical transmission potential of COVID-19 infection in nine pregnant women: a retrospective review of medical records. Lancet. (2020) 395:809–15. 10.1016/S0140-6736(20)30360-3 32151335 PMC7159281

[24] Zeng L Xia S Yuan W Yan K Xiao F Shao J. Neonatal early-onset infection with SARS-CoV-2 in 33 neonates born to mothers with COVID-19 in Wuhan, China. JAMA Pediatr. (2020) 174:722–5. 10.1001/jamapediatrics.2020.0878 32215598 PMC7099530

[25] Dumitriu D Emeruwa UN Hanft E Liao GV Ludwig E Walzer L. Outcomes of neonates born to mothers with severe acute respiratory syndrome coronavirus 2 infection at a large medical center in New York City. JAMA Pediatr. (2020) e204298. 10.1001/jamapediatrics.2020.4298.[Epub ahead of print]. PMC7551222 33044493

[26] Gheblawi M Wang K Viveiros A Nguyen Q Zhong JC Turner AJ. Angiotensin-converting enzyme 2: SARS-CoV-2 receptor and regulator of the renin-angiotensin system: celebrating the 20th anniversary of the discovery of ACE2. Circ Res. (2020) 126:1456–74. 10.1161/CIRCRESAHA.120.317015 32264791 PMC7188049

[27] Shi Y Wang Y Shao C Huang J Gan J Huang X. COVID-19 infection: the perspectives on immune responses. Cell Death Differ. (2020) 27:1451–4. 10.1038/s41418-020-0530-3 32205856 PMC7091918

[28] Xu Z Shi L Wang Y Zhang J Huang L Zhang C. Pathological findings of COVID-19 associated with acute respiratory distress syndrome. Lancet Respir Med. (2020) 8:420–2. 10.1016/S2213-2600(20)30076-XQ18 32085846 PMC7164771

[29] Lawrensia S Henrina J Wijaya E Suciadi LP Saboe A Cool CJ Pediatric inflammatory multisystem syndrome temporally associated with SARS-CoV-2: a new challenge amid the pandemic. SN Compr Clin Med. (2020) 2:2077–85. 10.1007/s42399-020-00602-8 PMC7578591 33106783

[30] Volpi S Naviglio S Tommasini A Covid-19 e risposta immune. Med Bambino. (2020) 39:223–31.

[31] Dong Y Mo X Hu Y Qi X Jiang F Jiang Z Epidemiological characteristics of 2143 pediatric patients with 2019 coronavirus disease in China. Pediatrics. (2020) 145:e20200702 10.1542/peds.2020-0702 32179660

[32] Shekerdemian LS Mahmood NR Wolfe KK Riggs BJ Ross CE McKiernan CA. International COVID-19 PICU Collaborative. Characteristics and Outcomes of Children With Coronavirus Disease 2019 (COVID-19) Infection Admitted to US and Canadian Pediatric Intensive Care Units. JAMA Pediatr. (2020) 174:1–6. 10.1001/jamapediatrics.2020.1948 32392288 PMC7489842

[33] Cristiani L Mancino E Matera L Nenna R Pierangeli A Scagnolari C Will children reveal their secret? The coronavirus dilemma. Eur Respir J. (2020) 55:2000749 10.1183/13993003.00749-2020 32241833 PMC7113798

[34] Saheb Sharif-Askari N Saheb Sharif-Askari F Alabed M Temsah MH Al Heialy S Hamid Q. Airways expression of SARS-CoV-2 receptor, ACE2, and TMPRSS2 is lower in children than adults and increases with smoking and COPD. Mol Ther Methods Clin Dev. (2020) 18:1–6. 10.1016/j.omtm.2020.05.013 32537478 PMC7242205

[35] Xie X Chen J Wang X Zhang F Liu Y. Age- and gender-related difference of ACE2 expression in rat lung. Life Sci. (2006) 78:2166–71. 10.1016/j.lfs.2006.09.028 16303146 PMC7094566

[36] Ortiz ME Thurman A Pezzulo AA Leidinger MR Klesney-Tait JA Karp PH. Heterogeneous expression of the SARS-Coronavirus-2 receptor ACE2 in the human respiratory tract. EBioMedicine. (2020) 60:102976. 10.1016/j.ebiom.2020.102976 32971472 PMC7505653

[37] Skarstein Kolberg E. ACE2, COVID19 and serum ACE as a possible biomarker to predict severity of disease. J Clin Virol. (2020) 126:104350. 10.1016/j.jcv.2020.104350 32283335 PMC7129721

[38] Foust AM McAdam AJ Chu WC Garcia-Pena P Phillips GS Plut D. Practical guide for pediatric pulmonologists on imaging management of pediatric patients with COVID-19. Pediatr. Pulmonol. (2020) 55:2213–24. 10.1002/ppul.24870 32462724 PMC7283678

[39] Oterino Serrano C Alonso E Andrés M Buitrago NM Pérez Vigara A Parrón Pajares M. Pediatric chest x-ray in covid-19 infection. Eur J Radiol. (2020) 131:109236. 10.1016/j.ejrad.2020.109236 32932176 PMC7448740

[40] Palabiyik F Kokurcan SO Hatipoglu N Cebeci SO Inci E. Imaging of COVID-19 pneumonia in children. Br J Radiol. (2020) 93:20200647. 10.1259/bjr.20200647 32730110 PMC7465849

[41] Duan YN Zhu YQ Tang LL Qin J. CT features of novel coronavirus pneumonia (COVID-19) in children. Eur Radiol. (2020) 30:4427–33. 10.1007/s00330-020-06860-3 32291501 PMC7156230

[42] Liu M Song Z Xiao K. High-resolution computed tomography manifestations of 5 pediatric patients with 2019 Novel Coronavirus. J Comp Assist Tomogr. (2020) 44:311–3. 10.1097/RCT.0000000000001023 32217900 PMC7228449

[43] Xia W Shao J Guo Y Peng X Li Z Hu D. Clinical and CT features in pediatric patients with COVID-19 infection: different points from adults. Pediatr Pulmonol. (2020) 55:1169–74. 10.1002/ppul.24718 32134205 PMC7168071

[44] Pan Y Guan H Zhou S Wang Y Li Q Zhu T. Initial CT findings and temporal changes in patients with the novel coronavirus pneumonia (2019-nCoV): a study of 63 patients in Wuhan, China. Eur Radiol. (2020) 30:3306–9. 10.1007/s00330-020-06731-x 32055945 PMC7087663

[45] Chen Z Fan H Cai J Li Y Wu B Hou Y. High-resolution computed tomography manifestations of COVID-19 infections in patients of different ages. Eur J Radiol. (2020) 126:108972. 10.1016/j.ejrad.2020.108972 32240913 PMC7102649

[46] Zheng F Liao C Fan QH Chen HB Zhao XG Xie ZG. Clinical characteristics of children with coronavirus disease 2019 in Hubei, China. Curr Med Sci. (2020) 40:275–80. 10.1007/s11596-020-2172-6 32207032 PMC7095065

[47] Sun D Li H Lu XX Xiao H Ren J Zhang FR. Clinical features of severe pediatric patients with coronavirus disease 2019 in Wuhan: a single center's observational study. World J Pediatr. (2020) 16:251–9. 10.1007/s12519-020-00354-4 32193831 PMC7091225

[48] Denina M Scolfaro C Silvestro E Pruccoli G Mignone F Zoppo M. Lung ultrasound in children with COVID-19. Pediatrics. (2020) 146:e20201157. 10.1542/peds.2020-1157 32317309

[49] Musolino AM Supino MC Buonsenso D Ferro V Valentini P Magistrelli A. Lung ultrasound in children with COVID-19: preliminary findings. Ultrasound Med Biol. (2020) 46:2094–8. 10.1016/j.ultrasmedbio.2020.04.026 32409232 PMC7196401

[50] Allinovi M Parise A Giacalone M Amerio A Delsante M Odone A. Lung ultrasound may support diagnosis and monitoring of COVID-19 pneumonia. Ultrasound Med Biol. (2020) 46:2908–17. 10.1016/j.ultrasmedbio.2020.07.018 32807570 PMC7369598

[51] College of Paediatrics and Child Health COVID-19: Guidance for Paediatric Services. (2020). Available online at: https://www.rcpch.ac.uk/resources/covid-19-guidance-paediatric-services (accessed October 1, 2020).

[52] Shen KL Yang YH Jiang RM Wang TY Zhao DC Jiang Y. Updated diagnosis, treatment and prevention of COVID-19 in children: experts' consensus statement (condensed version of the second edition). World J Pediatr. (2020) 16:232–9. 10.1007/s12519-020-00362-4 32333248 PMC7180653

[53] Fang L Karakiulakis G Roth M. Are patients with hypertension and diabetes mellitus at increased risk for COVID-19 infection? Lancet Respir Med. (2020) 8:e21. 10.1016/S2213-2600(20)30116-8 32171062 PMC7118626

[54] Cardinale F Ciprandi G Barberi S Bernardini R Caffarelli C Calvani M Consensus statement of the Italian society of pediatric allergy and immunology for the pragmatic management of children and adolescents with allergic or immunological diseases during the COVID-19 pandemic. Ital J Pediatr. (2020) 46:84 10.1186/s13052-020-00843-2 32546234 PMC7296524

[55] Yamamoto L Santos EHD Pinto LS Rocha MC Kanunfre KA Vallada MG. SARS-CoV-2 infections with emphasis on pediatric patients: a narrative review. Rev Inst Med Trop São Paulo. (2020) 62:e65. 10.1590/s1678-9946202062065 32901762 PMC7477958

[56] Oualha M Bendavid M Berteloot L Corsia A Lesage F Vedrenne M. Severe and fatal forms of COVID-19 in children. Arch Pediatr. (2020) 27:235–8. 10.1016/j.arcped.2020.05.010 32518045 PMC7269941

[57] Lyons C Callaghan M. The use of high-flow nasal oxygen in COVID-19. Anaesthesia. (2020) 75:843–7. 10.1111/anae.15073 32246843

[58] World Health Organization Clinical Management of Severe Acute Respiratory Infection (SARI) When COVID-19 Disease is Suspected. Interim Guidance. (2020). Available online at: www.who.int/publications-detail/clinical-management-of-severe-acute-http://www.who.int/publications-detail/clinical-management-of-severe-acute-respiratory-nfection-when-novel-coronavirus-(ncov)-infection-is-suspected (accessed October 1, 2020).

[59] Aliberti S Radovanovic D Billi F Sotgiu G Costanzo M Pilocane T. Helmet CPAP treatment in patients with COVID-19 pneumonia: a multicenter, cohort study. Eur Respir J. (2020) 56:2001935. 10.1183/13993003.01935-2020 32747395 PMC7397948

[60] Naja M Wedderburn L Ciurtin C. COVID-19 infection in children and adolescents. Br J Hosp Med. (2020) 81:1–10. 10.12968/hmed.2020.0321 32845750

[61] Chan JF Yao Y Yeung ML Deng W Bao L Jia L. Treatment with Lopinavir/Ritonavir or Interferon-β1b improves outcome of MERS-CoV infection in a nonhuman primate model of common marmoset. J Infect Dis. (2015) 212:1904–13. 10.1093/infdis/jiv392 26198719 PMC7107395

[62] Li H Wang YM Xu JY Cao B Potential antiviral therapeutics for 2019 Novel Coronavirus. Zhonghua Jie He He Hu Xi Za Zhi. (2020) 43:E002 10.3760/cma.j.issn.1001-0939.2020.0002 32023685

[63] Wang M Cao R Zhang L Yang X Liu J Xu M. Remdesivir and chloroquine effectively inhibit the recently emerged novel coronavirus (2019-nCoV) *in vitro*. Cell Res. (2020) 30:269–71. 10.1038/s41422-020-0282-0 32020029 PMC7054408

[64] Lu H. Drug treatment options for the 2019-new coronavirus (2019-nCoV). Biosci Trends. (2020) 14:69–71. 10.5582/bst.2020.01020 31996494

[65] Uddin M Mustafa F Rizvi TA Loney T Suwaidi HA Al-Marzouqi AHH. SARS-CoV-2/COVID-19: viral genomics, epidemiology, vaccines, and therapeutic interventions. Viruses. (2020) 12:526. 10.3390/v12050526 32397688 PMC7290442

[66] Poddighe D Aljofan M. Clinical evidences on the antiviral properties of macrolide antibiotics in the COVID-19 era and beyond. Antivir Chem Chemother. (2020) 28:2040206620961712. 10.1177/2040206620961712 32972196 PMC7522830

[67] Jones VG Mills M Suarez D Hogan CA Yeh D Segal JB. COVID-19 and Kawasaki Disease: novel virus and novel case. Hosp Pediatr. (2020) 10:537–40. 10.1542/hpeds.2020-0123 32265235

[68] Akca UK Kesici S Ozsurekci Y Aykan HH Batu ED Atalay E. Kawasaki-like disease in children with COVID-19. Rheumatol Int. (2020) 40:2105–15. 10.1007/s00296-020-04701-6 32936318 PMC7492688

[69] Stone JH Frigault MJ Serling-Boyd NJ Fernandes AD Harvey L Foulkes AS. Efficacy of Tocilizumab in patients hospitalized with Covid-19. N Engl J Med. (2020) 10.1056/NEJMoa2028836.[Epub ahead of print]. 33085857 PMC7646626

[70] Banday AZ Vignesh P. Use of tocilizumab in multisystem inflammatory syndrome in children associated with severe acute respiratory syndrome coronavirus 2. J Pediatr. (2020). 10.1016/j.jpeds.2020.09.054. [Epub ahead of print]. 32979386 PMC7587068

